# Risks and Protective Factors Associated With Mental Health Symptoms During COVID-19 Home Confinement in Italian Children and Adolescents: The #Understandingkids Study

**DOI:** 10.3389/fped.2021.664702

**Published:** 2021-06-11

**Authors:** Salvatore Oliva, Giusy Russo, Renata Gili, Luigi Russo, Antonio Di Mauro, Alessandra Spagnoli, Danilo Alunni Fegatelli, Maria Romani, Anna Costa, Silvio Veraldi, Filippo Manti

**Affiliations:** ^1^Maternal and Child Health Department, Sapienza – University of Rome, Rome, Italy; ^2^Primary Care, ASL CN1, Savigliano, Italy; ^3^GIMBE Foundation, Bologna, Italy; ^4^Department of Biomedical Science and Human Oncology, University “Aldo Moro”, Bari, Italy; ^5^Department of Public Health and Infectious Diseases, Sapienza – University of Rome, Rome, Italy; ^6^Department of Human Neuroscience, Sapienza – University of Rome, Rome, Italy; ^7^Service for Neurodevelopmental Disorders, University Campus Bio-Medico, Rome, Italy

**Keywords:** COVID-19, pediatrics, mental health, home confinement, neuropsychiatric disorders

## Abstract

**Objective:** To identify risk and protective factors for mental health symptoms associated with lifestyle changes caused by home confinement in pediatric subjects and in children and adolescents with a neuropsychiatric disorder.

**Study design:** This was a prospective, cross-sectional study conducted from May 10 to May 31, 2020. Two online anonymous surveys were developed: population-based and clinical-based (children with neuropsychiatric disorders). Outcomes included emotional and behavioral symptoms, as assessed by psychometric scales (BPSC, PPSC, PSC, CES-DC and SCARED, respectively), and lifestyle changes during home confinement (i.e., physical activity, screen time, home schooling, reading).

**Results:** The sample included 9,688 pediatric subjects, and 289 children and adolescents with a neuropsychiatric disorder. The presence of siblings was a protective factor in all ages. In pre- and school children: male sex, a diagnosis of autism, residency in highly affected areas, high parental educational level or job loss, and screen time (>2 h/day) were risk factors. Physical activity, home-schooling, reading, talking with other people were protective factors. Residency in highly affected areas, a diagnosis of mood disorder, parental job loss, and screen time, were associated with a worsening of the depressive symptoms, whereas physical activity, talking with other people, playing with parents were protective activities. Screen time was also a risk factor for anxiety symptoms, while physical activity, reading and talking with other people were protective factors.

**Conclusions:** This study identified risk and protective factors for mental health symptoms associated with lifestyle changes caused by COVID-19 home confinement to promote mental well-being in pediatrics during pandemic times.

## Introduction

During the coronavirus disease 2019 (COVID-19) pandemic, most national governments have temporarily imposed severe disease containment measures to prevent the spread of the infection. In Italy, one of the earlier and most affected European countries, a rigid home confinement was enforced from March 9, 2020 to May 4, 2020 (1). This measure led to implementation of home working, closures of schools, and prohibition of leaving houses for unnecessary activities. Despite being effective in flattening the epidemic curve, prolonged home confinement may have remarkable consequences on the population, especially in pediatric age (2). Evidence suggests that when children are out of school, they have substantial lifestyle changes such as less physical activity, longer screen time, irregular sleep patterns, and less favorable diets (3, 4). During home confinement, such negative changes can easily worsen, considering the prohibition of outdoor activities and the lack of peer interaction. Moreover, highly vulnerable groups, like children and adolescents with a neuropsychiatric disorder, might be deeply affected with consequent increase in anxiety and behavioral problems. Understanding children and adolescents' lives and emotions is essential to properly address their mental health needs and prevent psychological consequences.

The interaction between changes in daily habits and psychosocial stress in children and adolescents needs to be more investigated. Thus, we proposed that a better understanding of the psychological impact and lifestyle changes caused by home confinement on the Italian pediatric population, and on children and adolescents with a neuropsychiatric disorder, might contribute to identify protective and risk factors for mental health symptoms during the COVID-19 pandemic.

## Methods

### Participants and Procedures

This study collected a convenience sample of Italian children, immediately after the end of home confinement and national lockdown due to the Covid-19 outbreak. Data for this study were collected from May 10 to May 31, 2020, by using a survey distributed through a specific online platform. All families with at least one child <18 years and having access to internet and/or social media were eligible to participate. This project was advertised by a media sensitization campaign with the hashtag #understandingkids on social media to shortly collect a large sample.

Parents were asked to fill out an age-specific questionnaire for each household child. Specific questions for both parents and children were considered for participants ≥6 years, while only the parent's section was required for younger kids. The statement “I permit my child to participate in the survey” was presented to the guardian before the survey. Data were completely anonymized since no personally identifiable information was required. A second sample of children and adolescents with a neuropsychiatric disorder, followed by the Child Neurology and Psychiatry Unit at the Department of Human Neuroscience at Sapienza University of Rome, were collected during the study period. All families with a child affected by a neuropsychiatric disorder who have contacted the Unit during the Home Confinement were asked to participate. A separate online platform was created to collect data from this participants group. Parents filled out the same survey of the general population, except for a specific question on the neuropsychiatric diagnosis.

### Measures

The questionnaire includes 22 items measuring participants' demographics (both in children and parents), and children and adolescent's daily habits before and during the lockdown.

### Demographics Covariates

Demographic data for parents included: age range (<30, 30–34, 35–39, 40–44, >45), sex, educational level, employment status (including job loss due to the outbreak), residency, nationality, number of children, and civil status. Information on the other parent was also required regardless of the civil status. While in children the following demographics features were considered: age range (<1, 1–2, 3–5, 6–10, 11–13, 14–18 years), sex, number of siblings, and schooling activity (hours/weekly). The known diagnosis was recorded for subjects affected by a neuropsychiatric disorder.

### Lifestyles Changes

Daily habits were collected by asking parents about the frequency at which their children spent time before and during the lockdown in the following activities: (1) reading a book or playing with parents; (2) physical activity; (3) home-schooling; (4) reading or playing alone; (5) using social media; (6) interacting with other people by video chat, or phone; (7) gaming with a notebook, smartphone or other devices; (8) gaming with other people; (9) watching television; (10) watching video, movies or tv-series on any device; (11) talking with other people in person. The first three items (reading a book, playing with children, and physical activity) were measured using a 5-point weekly scale, while in the remaining a 5-point daily scale was preferred.

### Emotional and Behavior Assessments

Three common age-standardized pediatric primary care instruments were considered for the emotional and behavior assessments: the Baby Pediatric Symptom Checklist (BPSC), for infants (0–12 months); the Preschool Pediatric Symptom Checklist (PPSC), for pre-school children (1–6 years); and the Pediatric Symptom Checklist (PSC), for school children and adolescents (≥6 years) (5–7). These scores were collected and calculated asking parents about the emotional and behavioral status of their children before and during the lockdown. The BPSC score was also calculated by dividing it into three sub-scales including inflexibility, irritability, and routine. In children from 6 to 18 years, together with the PSC scale other two scores were considered to assess depressive and anxiety symptoms during the home confinement: the Center for Epidemiological Studies Depression Scale for Children (CES-DC), and the Screen for Child Anxiety Related Disorders (SCARED) (8, 9). Since the number of answers and the time spent for the survey increased by using three scores, only the effect during (and not before) the home confinement was inquired aged 6 years and above. Children and adolescents were invited to participate in answering questions with their parents while filling out the survey. Indeed, at the end of each questionnaire, questions specifically inquiring children about their feeling toward the epidemic (i.e., optimism about the epidemic or whether they worried about getting infected by COVID-19) were included. The complete survey is available in the Appendix. A mandatory answering option, which forces the participants to answer each question in order to complete the survey, was used.

### Statistical Analysis

Demographic characteristics of the study population were described using absolute and percent frequencies. A multiple linear regression model was used to evaluate the effect of demographics on the BPSC score. A model for each BPSC sub-score (inflexibility, irritability, routine) was defined considering the change from baseline (before lockdown) as the outcome, the baseline BPSC score value as adjustment covariate and all demographics characteristics as covariates.

A mixed-effect model was applied to estimate the effect of demographics and lifestyle changes on the PPSC score. This model included all demographic variables, time (before and during lockdown), the possible two-by-two interactions time and demographic variables, the autism diagnosis, and most age-significant lifestyle covariates. A subject-specific random intercept was used to account for dependence between repeated measurements on the same subject. Lifestyle variables were included in the model as baseline and as changes from the baseline in order to distinguish the cross-sectional and the longitudinal impact they have on PPSC score.

A multiple regression model was used to estimate the effect of demographic and lifestyles changes on PSC, CES-DC and SCARED in children ≥ 6 years. A model for each score was defined including all demographic characteristics, the presence of a neuropsychiatric disorder and the lifestyle changes categorized with respect to time.

Model selection was performed by stepwise procedure based on the Akaike Information Criterion (AIC). All analyses were performed using R version 4.0.0 software. The study protocol was defined in accordance with the Declaration of Helsinki and approved by the local Ethical Committee.

## Results

The survey on the general population was completed by 6,870 parents for a total of 9,688 children and adolescents (5,066 males, 52.3%), while the survey for families of subjects with neuropsychiatric disorders collected 255 answers, for a total of 289 participants (188 males, 65.1%). Table 1 shows the clinical and demographic characteristics of both groups.

**Table 1:** Characteristics of the study participants.

**Characteristics**	**General population No. (%)**	**Neuropsychiatric population No. (%)**
	9,688	289
**Sex**		
Male	5,066 (52.3)	188 (65.1)
Female	4,622 (47.7)	101 (34.9)
**Age**		
<1 year	860 (8.9)	0 (0.0)
1–2 years	2,707 (27.9)	33 (11.4)
3–5 years	3,695 (38.1)	33 (11.4)
6–10 years	1,576 (16.3)	66 (22.8)
11–13 years	629 (6.5)	80 (27.7)
14–18 years	221 (2.3)	77 (26.6)
**Cohabiting siblings**		
Yes	5,656 (58.4)	165 (57.1)
No	4,032 (41.6)	124 (42.9)
**Siblings, No**.		
0	4,032 (41.6)	124 (42.9)
1	4,990 (51.5)	127 (43.9)
2	597 (6.2)	31 (10.7)
≥3	69 (0.7)	7 (2.4)
**Neuropsychiatric disorders**		
Anxiety disorder	NA	33 (11.4)
ADHD	NA	36 (12.5)
Learning disability	NA	98 (33.9)
Mood disorder	NA	20 (6.9)
ASD	NA	80 (27.7)
OCD	NA	22 (7.6)
**Residency**		
Northern Italy	4,892 (50.5)	8 (2.8)
Central Italy	2,205 (22.8)	243 (84.1)
Southern Italy	2,591 (26.7)	38 (13.1)
**Parental age**		
<40 years	2,899 (42.4)	60 (21.3)
≥40 years	3,937 (57.6)	222 (78.7)
**Parental educational level**		
Degree	5,182 (72.6)	175 (60.8)
No degree	1,955 (27.4)	113 (39.2)

*ADHD, attention-deficit hyperactivity disorder; ASD, autism spectrum disorder; OCD, obsessive compulsive disorder; NA, not available*.

Remarkable lifestyle changes during the home confinement were observed in all participants of the two groups. Supplementary Figures 1–3 summarizes these findings in the different ages.

In children <1 year, 75.5% of subjects (649/860) from the general population had a positive BPSC test during the home confinement. An increase of 35% was observed from the period before the lockdown.

In pre-school children, the PPSC was above the threshold of positivity in 73.3% of normal subjects (4,695/6,402) and 80.3% (53/66) of children with a neuropsychiatric disorder, respectively, with an increase of 68 and 3.9% in both groups.

In school children and adolescents, 40.5% (983/2,426), 60% (1,457/2,426) and 42.4% (1,029/2,426) of the general population and 31.8% (71/223), 66.4% (148/223), and 38% (85/223) of participants with a neuropsychiatric disorder reported scores above cut-offs for clinically relevant symptoms on PSC, CES-DC and SCARED scales, respectively.

Tables 2, 3 present estimates from three regression models for BPSC, PPSC, PSC, SCARED, and CES-DC positivity (scores above the cut-off for clinically relevant symptoms) according to different age ranges.

**Table 2:** Demographic factors and lifestyle changes associated with mental health symptoms as assessed by age-specific psychometric tests: results from the multiple linear regression (BPSC) and the mixed-effect model (PPSC).

	**Estimate**	**SE**	***p*-value**
**Infants (0–12 months): emotional and behavioral symptoms (BPSC)**			
**Inflexibility**			
Siblings	−1.27213	0.22084	<0.001
**Irritability**			
Siblings	−0.77485	0.16961	<0.001
**Routine**			
Siblings	−0.54121	0.17503	0.002
**Pre-school children (1–6 years): emotional and behavioral symptoms**			
**(PPSC)**			
**Demographic characteristic**			
Male sex	0.881359	0.1758687	<0.001
Residency in highly affected area (Northern Italy)	0.663767	0.1798896	<0.001
Graduated parents	0.515992	0.1889927	0.006
ASD diagnosis	7.004670	0.8925715	<0.001
ASD diagnosis*Time	2.046034	0.9040033	0.02
Siblings	−0.499486	0.1764746	0.004
Parental job loss (both parents)*Time	1.097493	0.2863400	<0.001
**Lifestyle changes**			
Gaming with electronic devices alone at baseline >2 h/day	1.318075	0.3150962	<0.001
Increase in gaming with electronic devices alone during lockdown	2.330209	0.1836252	<0.001
Watching TV at baseline >2 h/day	1.703912	0.1563640	<0.001
Increase in watching TV during lockdown	2.395643	0.1576004	<0.001

*BPSC, baby pediatric symptom checklist; PPSC, preschool pediatric symptom checklist; SE, standard error; ASD, autism spectrum disorder*.

**Table 3:** Demographic factors and lifestyle changes associated with mental health symptoms as assessed by age-specific psychometric tests: results from the Multiple regression model.

	**Estimate**	**SE**	***p*-value**
**Emotional and behavioral symptoms (PSC)**			
**Demographic characteristic**			
Male sex	1.5902	0.5512	0.004
Residency in red zones (Northern Italy)	1.9277	0.5644	<0.001
Anxiety disorders	−6.7998	1.8959	<0.001
Learning disabilities	−5.5632	1.3344	<0.001
**Lifestyle changes**			
Physical activity (>once/week)	−5.7980	0.9179	<0.001
Watching video or TV series >2 h/day	3.0612	0.8153	<0.001
Home schooling >2 h/day	−2.7929	0.5561	<0.001
Using social media/chat >2 h/day	2.4173	0.6946	<0.001
Reading >2 h/day	−2.4683	0.8396	0.003
Gaming with electronic devices alone >2 h/day	1.8242	0.8153	0.02
Watching TV >2 h/day	1.7682	0.7521	0.02
Talking with other people in person >2 h/day	−1.8300	0.5584	0.001
**Depressive symptoms (CES-DC)**			
**Demographic characteristic**			
Residency in highly affected area (Northern Italy)	1.2065	0.3991	0.002
Parental job loss (both parents)	1.6837	0.5699	0.003
Mood disorders	6.3488	2.0975	0.002
**Lifestyle changes**			
Physical activity (≥once/week)	−3.0091	0.6451	<0.001
Watching video or TV series >2 h/day	1.8899	0.4806	<0.001
Using chat or social media >2 h/day	1.6648	0.5375	0.002
Playing with parents >once/week	−1.0478	0.4094	0.01
Talking with other people in person >2 h/day	−1.3564	0.4046	<0.001
**Anxiety symptoms (SCARED)**			
**Demographic characteristic**			
Siblings	−1.4180	0.6559	0.03
Learning disabilities	−6.2683	1.5289	<0.001
**Lifestyle changes**			
Physical activity (>once/week)	−4.3827	1.0762	<0.001
Watching video or TV series >2 h/day	2.3348	0.9437	0.01
Using social media/chat >2 h/day	2.7882	0.7913	<0.001
Reading >2 h/day	−2.0005	0.9858	0.04
Talking with other people in person >2 h/day	−1.7701	0.6569	0.007

*PSC, pediatric symptom checklist; CES-DC, Center for Epidemiological Studies Depression Scale; SCARED, screen for child anxiety related disorders*.

The presence of siblings was a protective factor for each BPSC sub-score (*p* < 0.001). When examining PPSC in pre-school age, several demographic variables were predictors of a positive score: male, diagnosis of autism, residency in a highly affected area (Northern Italy), high parental educational level (both graduated), parental job loss because of the pandemic (*p* < 0.01); while the presence of siblings was a protective factor (*p* < 0.005). After adjusting variables for the changes during the lockdown, the diagnosis of autism spectrum disorder and parental job loss were associated with higher worsening of test scores (*p* < 0.05). With regards to lifestyle changes, the use of smartphone >2 h/day, watching television >2 h/day, or their increase during the lockdown were independently predictors of a clinical score above the normal cut-off on the PPSC.

In school children and adolescents, male and residency in Northern Italy were predictive of a PSC clinical score during the lockdown, while a diagnosis of anxiety disorders or learning disabilities were inversely associated (*p* < 0.001). Time (>2 h) spent on social media or chat, watching television or tv series/movies on digital devices, and using smartphone were risk factors, while physical activity, home-schooling, reading a book, talking with other people were considered protective (*p* < 0.05).

When examining depressive symptoms through CES-DC, children and adolescents with residency in Northern Italy, with a diagnosis of mood disorders, and with the loss of parental job were more likely presenting clinical scores (*p* < 0.005). Lifestyle changes more associated with depressive symptoms were time spent on social media/chat, watching tv series/movies on digital devices (*p* < 0.001), whereas physical activity, talking with other people, playing with parents were protective activities (*p* < 0.05).

Lastly, in evaluating anxiety as per SCARED results, no demographic characteristic was associated with clinical scores, but the presence of siblings and the diagnosis of learning disabilities were inversely associated (*p* < 0.001). Time spent on social media/chat and watching tv series/movies on digital devices were risk factors (*p* < 0.001), whereas physical activity, reading and talking with other people were protective factors (*p* < 0.05).

**Figure 1:**
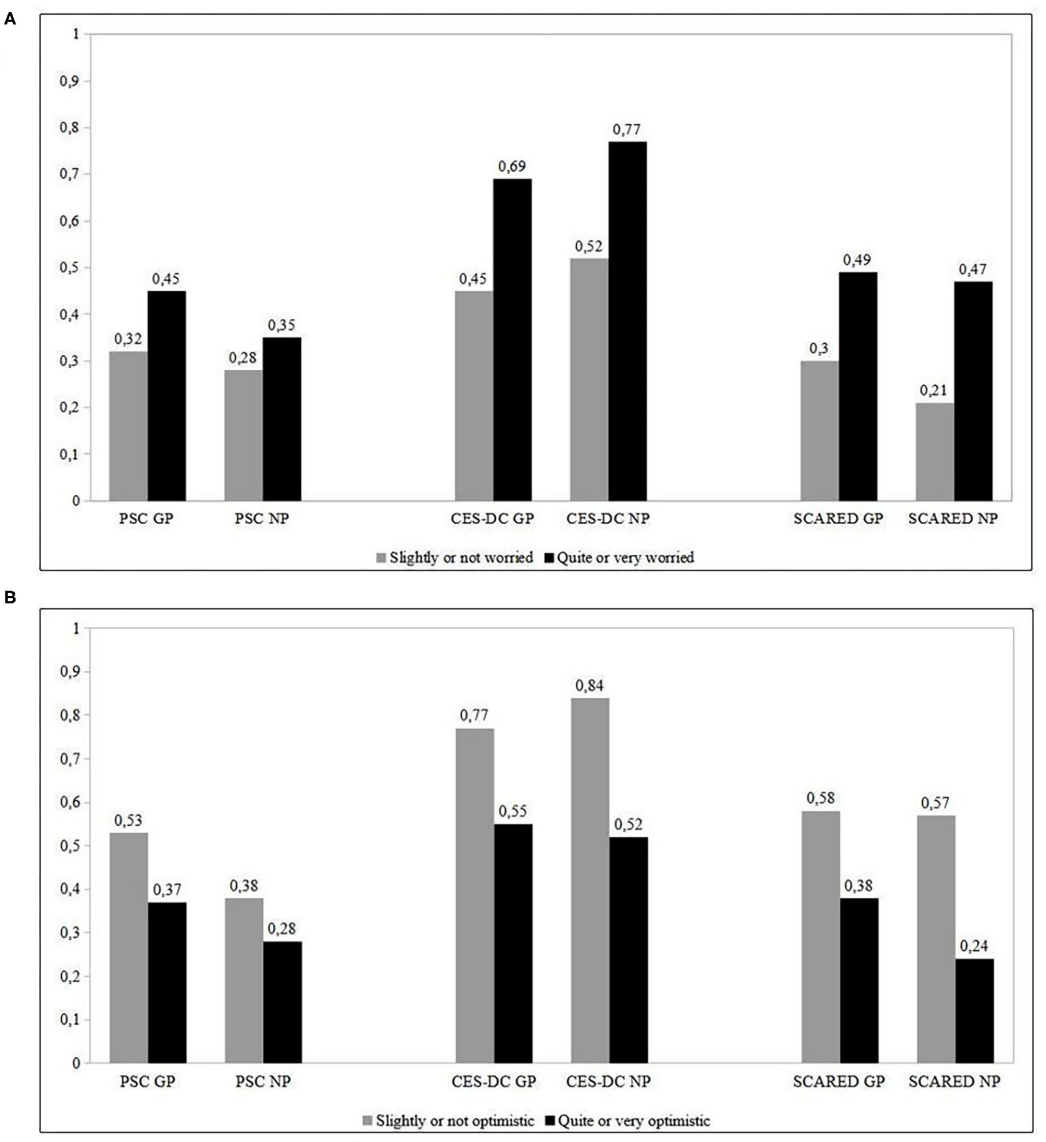
**(A)** Rate of subjects positive to psychometric scores who were worried about being infected between the study groups. **(B)** Rate of subjects positive to psychometric scores who were optimistic about the pandemic between the study groups. GP, general population; NP, neuropsychiatric group.

In children and adolescents, answers about optimism toward the epidemic or worry about getting infected were also recorded. Overall, 63.4% (1,539/2,426) and 57.8% (129/223) were worried about the pandemic in the general population and in the group with a neuropsychiatric disorder, respectively. In subjects with positive psychometric scores among the two groups, lack of optimism or worry about getting infection were more frequently reported (Figure 1).

## Discussion

This study aimed to address the lifestyle changes and psychological effects caused by home confinement in the Italian pediatric population, and in children and adolescents with a neuropsychiatric disorder. The main findings highlight emotional and behavioral symptoms considerably increased in pediatric population during the national lockdown. Several demographic and clinical characteristics (male sex, residency in highly affected area, parental job loss, educational level of parents, and autism spectrum disorders diagnosis) seemed associated with a higher risk of psychological consequences. Moreover, since the enforced social isolation has generated remarkable lifestyle changes in all, several protective and risk factors were identified for mental health symptoms. The presence of siblings, the time spent for physical activity, talking with other people or reading, resulted protective factors, whereas using a smartphone, watching television/tv-series/movies on digital devices, or time spent on social media or chat, were risk factors in an age-specific manner.

These findings are important for several reasons. First, this study comprehensively addresses the psychological impact caused by a long home confinement (2 months) on children and adolescents, including those with a neuropsychiatric disorder. Higher frequencies of psychometric tests above the clinical cut-off were obtained in pre-school children (>70%) compared with school children and adolescents (40.5%). Overall, the available evidence addresses an increased rate of psychological problems during the containment measures (ranging from 20 to 40%), considering that the international prevalence of all pediatric mental disorders was 13.4% before the pandemic (10–13). A more severe course of mental disorders might be expected in the short- and long-term and need to be carefully monitored, especially in subjects with a pre-existing disorder (14, 15).

Second, the use of an extremely detailed survey without missing data allowed the identification of both protective and risk factors among demographic characteristics and lifestyle changes owing by home confinement.

The presence of siblings was the only protective factor for infants. This confirms the idea that positive sibling relationships may be protective to the development of psychological difficulties and maladjustment (16). Differently from what has been described, the male sex was a risk factor in pre-school children (10, 11, 17). Residency in Northern Italy, the most affected area in Italy, was another significant risk factor, thus confirming the higher incidence of mental illness symptoms among children residing in highly infected areas (10, 12). The loss of parental job, and graduated parents were both risk factors. Expectations of an undesirable economic development might determine negative behaviors among family members. Parents with higher educational levels usually have jobs that are compatible with remote work, thus they could be forced into the role of teachers of their kids, while working from home and coping with a higher stress burden.

Physical activity was the most important protective factor for all tests in school children and adolescents. A recent study has found that children and adolescents' physical activity was at very low level during the pandemic, but it considerably reduced child mental distress related to COVID-19 (12, 18). In Italy, rigid restrictions were enforced for 2 months, thus children living in urban areas and small apartments were more likely affected. The outdoor activities should be implemented to prevent such a reduction and related risks during containment measures.

This study also confirms previous evidence about the correlation between screen exposure and neuropsychiatric symptoms (19). Prolonged screen time activities were predictive of developing mental health symptoms during the home confinement in almost all ages. Although the onset of these symptoms is likely determined by multiple factors, limiting the screen exposure is of critical importance in maintaining mental well-being status in pediatric age. High-quality and time-limited screen programming, or co-viewing should be preferred and moderately implemented when children cannot interact with their peer. In particular, children with an autism spectrum disorder might be at higher risk of a digital device abuse during the lockdown, since this disorder often makes portable media highly appealing (20).

Home-schooling might also participate in increasing screen time exposure. However, in our analysis, home-schooling was a protective factor. School closure is one of the most disruptive forces in the COVID-19 era, but home-schooling helps to keep learning activities going on, while reducing stressors related to home confinement. Surely, home-learning should be a temporary solution because schools support social, emotional, and physical well-being. Since the real effect of school closure in curve mitigation has yet to be defined (21), a careful cost/effective analysis should be considered before taking any political decision.

This study also provides the first evidence that pre-school children with a diagnosis of autism spectrum disorder were at higher risk of symptom intensification during the lockdown. Conversely, subjects with an anxiety disorder or learning disability were less affected. Likely, they positively perceived home confinement, thus experiencing less psychological distress and more life satisfaction. A longer time spent with parents and siblings has possibly contributed. Moreover, home-schooling likely determined new forms of accommodation and personalization in learning.

Reading, playing with parents, and talking with other people were all confirmed as protective factors for mental health symptoms.

Lastly, the rate of psychometric tests above the clinical cut-off significantly differed when children and adolescents reported negative feelings toward the epidemic. This confirms the necessity of increasing positive communication to address children and adolescents' fears and concerns during home confinement (12).

### Limitations

Certain limitations must be acknowledged. Our data are cross-sectional and collected by an online survey, which limits the quality of answers and assessments of daily habits. However, this was the only way to efficiently collect data while respecting containment measures.

Although our sample was recruited from across all Italian regions, participants self-selected into this study via social media or internet, which may be prone to selection bias. This may limit the generalizability of our findings by potentially influencing comparisons. The statistical methods we applied in the different ages aimed to minimize these limitations. Finally, we cannot exclude that a proportion of children with neuropsychiatric disorders might also be included in the sample from general population, as our populations were recruited from two different scenarios (online survey available to all and clinical setting). This may have led to overestimate the results obtained from general populations. However, despite having two groups with unequal sizes, the number of participants well-represents the actual ratio between general population and children with a neuropsychiatric condition.

## Conclusions

Overall, our findings highlight that lifestyle changes caused by home confinement during the COVID-19 pandemic had a significant psychological impact on pediatric population. In particular, subjects with autism spectrum disorders seem the most affected. Encouraging physical activity or other positive behaviors (i.e., interacting with siblings or other people, reading, home-schooling), can reduce the psychological impact that children and adolescents may feel. Relieving children's distress by media entertainment negatively impacted the pediatric mental health. Effective measures to promote a positive psychological attitude toward the pandemic needs to be supported in childhood. At the beginning of the second wave in Europe, more attention should be paid to the children and adolescents' mental health status before adopting important containment measures, including school closure.

## Data Availability Statement

The raw data supporting the conclusions of this article will be made available by the authors, without undue reservation.

## Ethics Statement

The studies involving human participants were reviewed and approved by Policlinico Umberto I Ethics committee. Written informed consent to participate in this study was provided by the participants' legal guardian/next of kin.

## Author Contributions

SO, GR, SV, AD, and FM: conception and design of the study. LR, RG, AD, FM, MR, and AC: data collection. LR, AS, and DA: analysis and interpretation of data. SO, GR, SV, and FM: drafting the article. SO, MR, and AC: literature review. All authors critically revising the article, final approval of the manuscript, and have verified the collected data.

## Conflict of Interest

The authors declare that the research was conducted in the absence of any commercial or financial relationships that could be construed as a potential conflict of interest.

## Supplementary Material

Supplementary Figure 1 Daily habits before and during the home confinement in infants.

Supplementary Figure 2 Daily habits before and during the home confinement in pre-school children between the study groups. GP, general population; NP, neuropsychiatric group.

Supplementary Figure 3 Daily habits before and during the home confinement in school children and adolescents between the study groups. GP, general population; NP, neuropsychiatric group.

## Abbreviations

BPSC: Baby Pediatric Symptom Checklist
CES-DC: Center for Epidemiological Studies Depression Scale for Children
COVID-19: Coronavirus disease 2019
PPSC: Preschool Pediatric Symptom Checklist
PSC: Pediatric Symptom Checklist
SCARED: Screen for Child Anxiety Related Disorders.

## References

[1] DPCM. Ulteriori Disposizioni Attuative del Decreto-Legge 23 Febbraio 2020, n. 6, Recante Misure Urgenti in Materia di Contenimento e Gestione dell'emergenza Epidemiologica da COVID-19, Applicabili Sull'intero Territorio Nazionale. Italy (2020).

[2] Hawryluck L Gold WL Robinson S Pogorski S Galea S Styra R. SARS control and psychological effects of quarantine, Toronto, Canada. Emerg Infect Dis. (2004) 10:1206–12. 10.3201/eid1007.030703 15324539 PMC3323345

[3] Brazendale K Beets MW Weaver RG Pate RR Turner-McGrievy GM Kaczynski AT. Understanding differences between summer vs. school obesogenic behaviors of children: the structured days hypothesis. Int J Behav Nutr Phys Act. (2017) 14:100. 10.1186/s12966-017-0555-2 28747186 PMC5530518

[4] Wang G Zhang J Lam SP Li SX Jiang Y Sun W. Ten-year secular trends in sleep/wake patterns in Shanghai and Hong Kong school-aged children: a tale of two cities. J Clin Sleep Med. (2019) 15:1495–502. 10.5664/jcsm.7984 31596215 PMC6778342

[5] Sheldrick RC Henson BS Merchant S Neger EN Murphy JM Perrin EC. The Preschool Pediatric Symptom Checklist (PPSC): development and initial validation of a new social/emotional screening instrument. Acad Pediatr. (2012) 12:456–67. 10.1016/j.acap.2012.06.008 22921494 PMC3907071

[6] Sheldrick RC Henson BS Neger EN Merchant S Murphy JM Perrin EC. The baby pediatric symptom checklist: development and initial validation of a new social/emotional screening instrument for very young children. Acad Pediatr. (2013) 13:72–80. 10.1016/j.acap.2012.08.003 23092547 PMC3763819

[7] Jellinek MS Murphy JM Burns BJ. Brief psychosocial screening in outpatient pediatric practice. J Pediatr. (1986) 109:371–8. 10.1016/S0022-3476(86)80408-5 3734977

[8] Faulstich ME Carey MP Ruggiero L Enyart P Gresham F. Assessment of depression in childhood and adolescence: an evaluation of the Center for Epidemiological Studies Depression Scale for Children (CES-DC). Am J Psychiatry. (1986) 143:1024–7. 10.1176/ajp.143.8.1024 3728717

[9] Birmaher B Khetarpal S Brent D Cully M Balach L Kaufman J. The Screen for Child Anxiety Related Emotional Disorders (SCARED): scale construction and psychometric characteristics. J Am Acad Child Adolesc Psychiatry. (1997) 36:545–53. 10.1097/00004583-199704000-00018 9100430

[10] Xie X Xue Q Zhou Y Zhu K Liu Q Zhang J. Mental health status among children in home confinement during the coronavirus disease 2019 outbreak in Hubei Province, China. JAMA Pediatr. (2020) 174:898–900. 10.1001/jamapediatrics.2020.1619 32329784 PMC7182958

[11] Liu Q Zhou Y Xie X Xue Q Zhu K Wan Z. The prevalence of behavioral problems among school-aged children in home quarantine during the COVID-19 pandemic in china. J Affect Disord. (2020) 279:12550. 10.1016/j.jad.2020.10.008 33099056 PMC7543949

[12] Jiao WY Wang LN Liu J Fang SF Jiao FY Pettoello-Mantovani M. Behavioral and emotional disorders in children during the COVID-19 epidemic. J Pediatr. (2020) 221:264–6.e1. 10.1016/j.jpeds.2020.03.013 32248989 PMC7127630

[13] Polanczyk GV Salum GA Sugaya LS Caye A Rohde LA. Annual research review: a meta-analysis of the worldwide prevalence of mental disorders in children and adolescents. J Child Psychol Psychiatry. (2015) 56:345–65. 10.1111/jcpp.12381 25649325

[14] Simon NM Saxe GN Marmar CR. Mental health disorders related to COVID-19-related deaths. JAMA. (2020) 324:1493–4. 10.1001/jama.2020.19632 33044510 PMC11404536

[15] Ettman CK Abdalla SM Cohen GH Sampson L Vivier PM Galea S. Prevalence of depression symptoms in US adults before and during the COVID-19 pandemic. JAMA Netw Open. (2020) 3:e2019686. 10.1001/jamanetworkopen.2020.19686 32876685 PMC7489837

[16] Gass K Jenkins J Dunn J. Are sibling relationships protective? A longitudinal study. J Child Psychol Psychiatry. (2007) 48:167–75. 10.1111/j.1469-7610.2006.01699.x 17300555

[17] Oosterhoff B Palmer CA. Attitudes and psychological factors associated with news monitoring, social distancing, disinfecting, and hoarding behaviors among US adolescents during the coronavirus disease 2019 pandemic. JAMA Pediatr. (2020) 29:e201876. 10.1001/jamapediatrics.2020.1876 32597925 PMC7325067

[18] Zhang X Zhu W Kang S Qiu L Lu Z Sun Y. Association between physical activity and mood states of children and adolescents in social isolation during the COVID-19 epidemic. Int J Environ Res Public Health. (2020) 17:7666. 10.3390/ijerph17207666 33096659 PMC7589310

[19] Madigan S McArthur BA Anhorn C Eirich R Christakis DA. Associations between screen use and child language skills: a systematic review and meta-analysis. JAMA Pediatr. (2020) 174:665–75. 10.1001/jamapediatrics.2020.0327 32202633 PMC7091394

[20] Lane R Radesky J. Digital media and autism spectrum disorders: review of evidence, theoretical concerns, and opportunities for intervention. J Dev Behav Pediatr. (2019) 40:364–8. 10.1097/DBP.0000000000000664 30973425 PMC6579611

[21] Li Y Campbell H Kulkarni D. The temporal association of introducing and lifting non-pharmaceutical interventions with the time-varying reproduction number (R) of SARS-CoV-2: a modelling study across 131 countries. Lancet Infect Dis. (2020) 21:193–202. 10.1016/S1473-3099(20)30785-4 33729915 PMC7581351

