# Supplementary material for: Risks and Protective Factors Associated With Mental Health Symptoms During COVID-19 Home Confinement in Italian Children and Adolescents: The #Understandingkids Study
Source: Front Pediatr. 2021 Jun 11;9:664702. doi: 10.3389/fped.2021.664702 (PMC8225997; doi:10.3389/fped.2021.664702)
Supplement: Supplementary Figure 1 — Daily habits before and during the home confinement in infants. [file Image_1.pdf]

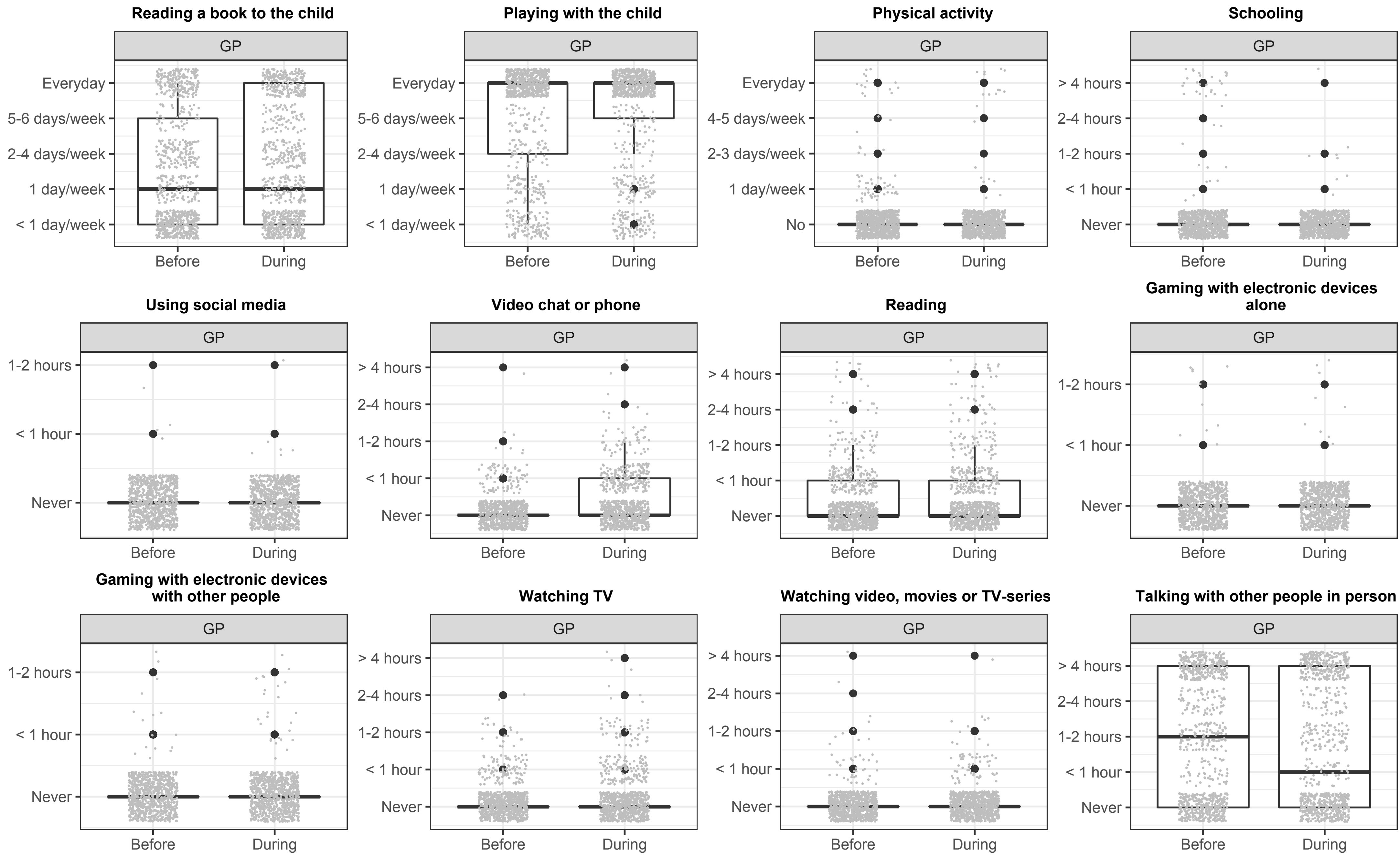
Reading a book to the child
GP
Everyday
5-6 days/week
2-4 days/week
1 day/week
< 1 day/week
Before
During
Playing with the child
GP
Everyday
5-6 days/week
2-4 days/week
1 day/week
< 1 day/week
Before
During
Physical activity
GP
Everyday
4-5 days/week
2-3 days/week
1 day/week
No
Before
During
Schooling
GP
> 4 hours
2-4 hours
1-2 hours
< 1 hour
Never
Before
During
Using social media
GP
1-2 hours
< 1 hour
Never
Before
During
Video chat or phone
GP
> 4 hours
2-4 hours
1-2 hours
< 1 hour
Never
Before
During
Reading
GP
> 4 hours
2-4 hours
1-2 hours
< 1 hour
Never
Before
During
Gaming with electronic devices alone
GP
1-2 hours
< 1 hour
Never
Before
During
Gaming with electronic devices with other people
GP
1-2 hours
< 1 hour
Never
Before
During
Watching TV
GP
> 4 hours
2-4 hours
1-2 hours
< 1 hour
Never
Before
During
Watching video, movies or TV-series
GP
> 4 hours
2-4 hours
1-2 hours
< 1 hour
Never
Before
During
Talking with other people in person
GP
> 4 hours
2-4 hours
1-2 hours
< 1 hour
Never
Before
During
