# Supplementary material for: Risks and Protective Factors Associated With Mental Health Symptoms During COVID-19 Home Confinement in Italian Children and Adolescents: The #Understandingkids Study
Source: Front Pediatr. 2021 Jun 11;9:664702. doi: 10.3389/fped.2021.664702 (PMC8225997; doi:10.3389/fped.2021.664702)
Supplement: Supplementary Figure 3 — Daily habits before and during the home confinement in school children and adolescents between the study groups. GP, general population; NP, neuropsychiatric group. [file Image_3.pdf]

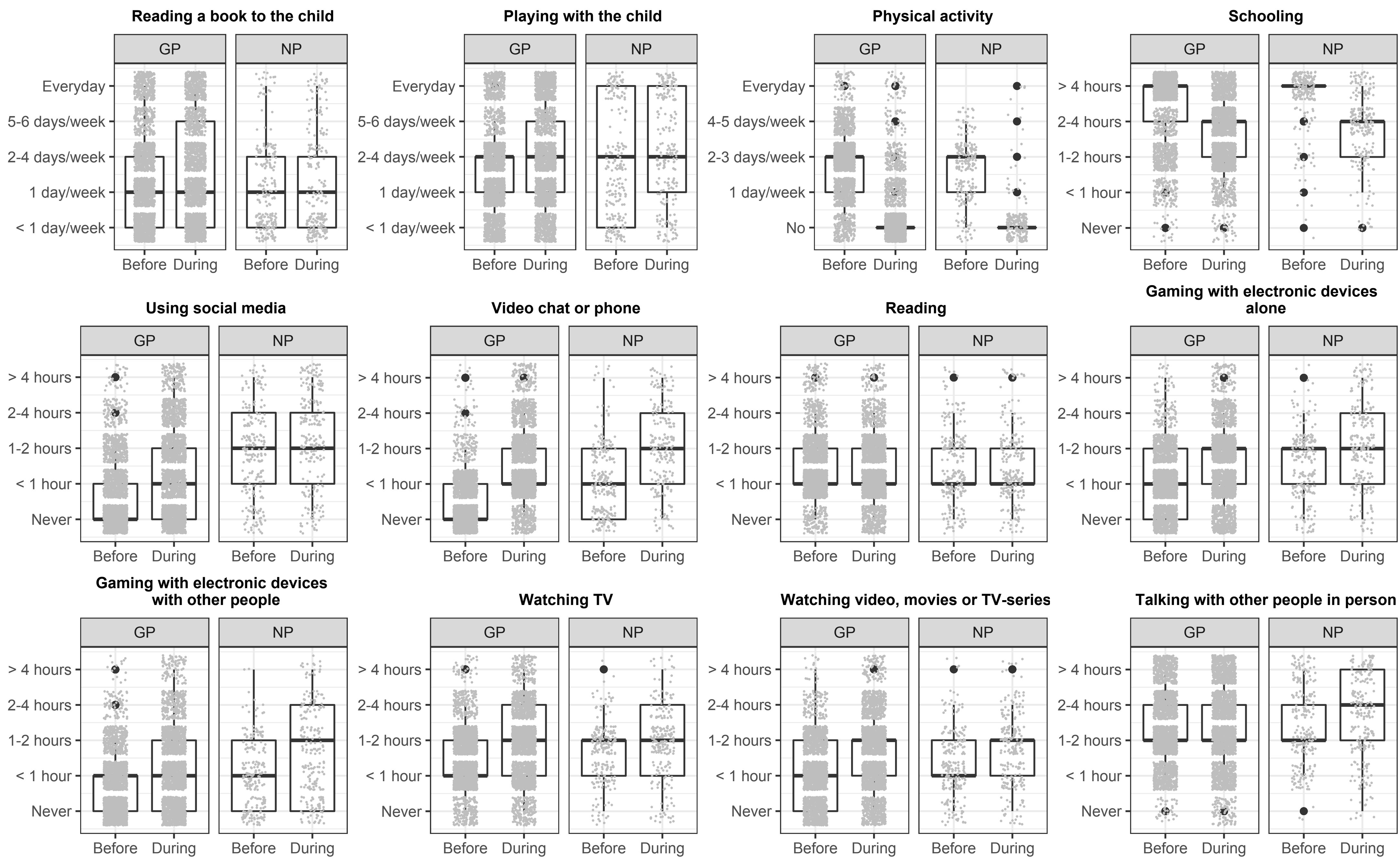
Reading a book to the child
Playing with the child
Physical activity
Schooling
Using social media
Video chat or phone
Reading
Gaming with electronic devices alone
Gaming with electronic devices with other people
Watching TV
Watching video, movies or TV-series
Talking with other people in person
GP
NP
Everyday
5-6 days/week
2-4 days/week
1 day/week
< 1 day/week
4-5 days/week
2-3 days/week
No
> 4 hours
2-4 hours
1-2 hours
< 1 hour
Never
Before
During
