# Supplementary material for: Risks and Protective Factors Associated With Mental Health Symptoms During COVID-19 Home Confinement in Italian Children and Adolescents: The #Understandingkids Study
Source: Front Pediatr. 2021 Jun 11;9:664702. doi: 10.3389/fped.2021.664702 (PMC8225997; doi:10.3389/fped.2021.664702)
Supplement: Supplementary file 4 [file Data_Sheet_1.docx]

**#UNDERSTANDINGKIDS**

**1.** Age of the parent completing the survey:

- <30 years old

- 30-34 years old

- 35- 39 years old

- 40 – 45 years old

- > 45 years old

**2.** Educational level

- Nothing

- Primary school diploma

- Secondary school diploma

- High School Diploma

- Technical / professional diploma

- Advanced study course of art, music, dance

- Graduation

- Master's degree

- Post-graduate degree

**3.** Working position:

- Part time worker

- Full-time worker

- Unemployed

- Unable to work

- Retired

**4.** Nationality

- Italian

- Other

**5.** If the nationality is different from the Italian one, specify (e.g. American)

**6.** Province of residence

- Agrigento

- Alessandria

- Ancona

- Aosta

- L'Aquila

- Arezzo

- Ascoli-Piceno

- Asti

- Avellino

- Bari

- Barletta-Andria-Trani

- Belluno

- Benevento

- Bergamo

- Biella

- Bologna

- Bolzano

- Brescia

- Brindisi

- Cagliari

- Caltanissetta

- Campobasso

- Carbonia Iglesias

- Caserta

- Catania

- Catanzaro

- Chieti

- Como

- Cosenza

- Cremona

- Crotone

- Cuneo

- Enna

- Fermo

- Ferrara

- Firenze

- Foggia

- Forli-Cesena

- Frosinone

- Genova

- Gorizia

- Grosseto

- Imperia

- Isernia

- La-Spezia

- Latina

Lecce

- Lecco

- Livorno

- Lodi

- Lucca

- Macerata

- Mantova

- Massa-Carrara

- Matera

- Medio Campidano

- Messina

- Milano

- Modena

- Monza-Brianza

- Napoli

- Novara

- Nuoro

- Ogliastra

- Olbia Tempio

- Oristano

- Padova

- Palermo

- Parma

- Pavia

- Perugia

- Pesaro-Urbino

- Pescara

- Piacenza

- Pisa

- Pistoia

- Pordenone

- Potenza

- Prato

- Ragusa

- Ravenna

- Reggio-Calabria

- Reggio-Emilia

- Rieti

- Rimini

- Roma

- Rovigo

- Salerno

- Sassari

- Savona

- Siena

- Siracusa

- Sondrio

- Taranto

- Teramo

- Terni

- Torino

- Trapani

- Trento

- Treviso

- Trieste

- Udine

- Varese

- Venezia

- Verbania

- Vercelli

- Verona

- Vibo-Valentia

- Vicenza

- Viterbo

**7.** Number of children

*The following questions are about the other parent.*

**8.** Is the other parent cohabiting?

- Yes

- No, because we're divorced

- No, because we're separated

- No, for business reasons

- No, for other reasons

**9.** Age of the other parent

- <30 years old

- 30-34 years old

- 35- 39 years old

- 40 – 45 years old

- > 45 years old

**10.** Educational level

- Nothing

- Primary school diploma

- Secondary school diploma

- High School Diploma

- Technical / professional diploma

- Advanced study course of art, music, dance

- Graduation

- Master's degree

- Post-graduate degree

**11.** Working position:

- Part time worker

- Full-time worker

- Unemployed

- Unable to work

- Retired

**12.** Did you lose your job during the lockdown?

-No, neither of them

- Yes, one

- Yes, both

**Child 1**

**1.** Sex

- Male

- Female

**2.** Age

- <1 year old

- 1-2 years old

- 3-5 years old

- 6-10 years old

- 11-13 years old

- 14-18 years old

**3.** Number of cohabiting siblings:

- nobody

- 1

- 2

- 3

- >3

**4.** Is the child in smart schooling during this period?

-Yes

- No

**5.** If yes, how many days a week?

-1

- 2

- 3

- 4

- 5

- 6

*In the following questions, indicate how often the following situations occurred before lockdown due to the COVID-19 outbreak and how often they occur during this period.*

**6.** You read a book with your child:

|  | <once/week | once/week | 2-4 times/ week | 5-6 times/ week | Every day |
| --- | --- | --- | --- | --- | --- |
| Before the lockdown |  |  |  |  |  |
| During the lockdown |  |  |  |  |  |

**7.** You play with your baby:

|  | <once/week | once/week | 2-4 times/ week | 5-6 times/ week | Every day |
| --- | --- | --- | --- | --- | --- |
| Before the lockdown |  |  |  |  |  |
| During the lockdown |  |  |  |  |  |

**8.** Your child practices physical activity:

|  | <once/week | once/week | 2-4 times/ week | 5-6 times/ week | Every day |
| --- | --- | --- | --- | --- | --- |
| Before the lockdown |  |  |  |  |  |
| During the lockdown |  |  |  |  |  |

**9.** Please indicate how many hours a day your child carried out the following activities BEFORE lockdown due to the COVID-19 pandemic.

|  | never | <1 hour/day | 1-2 hours/day | 2-4 hours/day | > 4 hours/day |
| --- | --- | --- | --- | --- | --- |
| Schooling |  |  |  |  |  |
| Using social media |  |  |  |  |  |
| Interacting with other people by video chat, or phone |  |  |  |  |  |
| Reading |  |  |  |  |  |
| Gaming with a notebook, smartphone or other devices alone |  |  |  |  |  |
| Gaming with notebook, smartphone or other devices with other people |  |  |  |  |  |
| Watching television |  |  |  |  |  |
| Watching video, movies or tv-series on any device |  |  |  |  |  |
| Talking with other people in person. |  |  |  |  |  |

**10.** Indicate how many hours a day your child carried out the following activities DURING lockdown due to the COVID-19 pandemic.

|  | never | <1 hour/day | 1-2 hours/day | 2-4 hours/day | > 4 hours/day |
| --- | --- | --- | --- | --- | --- |
| Schooling |  |  |  |  |  |
| Using social media |  |  |  |  |  |
| Interacting with other people by video chat, or phone |  |  |  |  |  |
| Reading |  |  |  |  |  |
| Gaming with a notebook, smartphone or other devices alone |  |  |  |  |  |
| Gaming with notebook, smartphone or other devices with other people |  |  |  |  |  |
| Watching television |  |  |  |  |  |
| Watching video, movies or tv-series on any device |  |  |  |  |  |
| Talking with other people in person. |  |  |  |  |  |

**11.**The child is followed by the Child Neurology and Psychiatry Unit for*:

- Autism Spectrum Disease

- Attention Deficit Hyperactivity Disorder (ADHD)

- Learning Disability

- Mood disorders

- Anxiety disorder

- Obsessive Compulsive Disorder

**Range 0-12 months**

*In the following questions indicate, for each behavior, how often it occurred in your child before lockdown due to the COVID-19 pandemic and how often it occurs during the lockdown.*

**1.** BEFORE the lockdown, your child:

|  | Not at all | Somewhat | Very Much |
| --- | --- | --- | --- |
| Did your child have a hard time being with new people? |  |  |  |
| Did your child have a hard time in new places? |  |  |  |
| Did your child have a hard time with change? |  |  |  |
| Did your child mind being held by other people? |  |  |  |
| Did your child cry a lot? |  |  |  |
| Did your child have a hard time calming down? |  |  |  |
| Was your child fussy or irritable? |  |  |  |
| Was it hard to comfort your child? |  |  |  |
| Was it hard to keep your child on a schedule or routine? |  |  |  |
| Was it hard to put your child to sleep? |  |  |  |
| Was it hard to get enough sleep because of your child? |  |  |  |
| Did your child have trouble staying asleep? |  |  |  |

**2.** DURING the lockdown, your child:

|  | Not at all | Somewhat | Very Much |
| --- | --- | --- | --- |
| Does your child have a hard time being with new people? |  |  |  |
| Does your child have a hard time in new places? |  |  |  |
| Does your child have a hard time with change? |  |  |  |
| Does your child mind being held by other people? |  |  |  |
| Does your child cry a lot? |  |  |  |
| Does your child have a hard time calming down? |  |  |  |
| Is your child fussy or irritable? |  |  |  |
| Is it hard to comfort your child? |  |  |  |
| Is it hard to keep your child on a schedule or routine? |  |  |  |
| Is it hard to put your child to sleep? |  |  |  |
| Is it hard to get enough sleep because of your child? |  |  |  |
| Does your child have trouble staying asleep? |  |  |  |

**Range 1-6 years**

*In the following questions indicate, for each behavior, how often it occurred in your child before lockdown due to the COVID-19 pandemic and how often it occurs during the lockdown.*

**1.** BEFORE the lockdown, your child:

|  | Not at all | Somewhat | Very Much |
| --- | --- | --- | --- |
| Did your child seem nervous or afraid? |  |  |  |
| Did your child seem sad or unhappy? |  |  |  |
| Did your child get upset if things are not done in a certain way? |  |  |  |
| Did your child have a hard time with change? |  |  |  |
| Did your child have trouble playing with other children? |  |  |  |
| Did your child break things on purpose? |  |  |  |
| Did your child fight with other children? |  |  |  |
| Did your child have trouble paying attention? |  |  |  |
| Did your child have a hard time calming down? |  |  |  |
| Did your child have trouble staying with one activity? |  |  |  |
| Was your child aggressive? |  |  |  |
| Was your child fidgety or unable to sit still? |  |  |  |
| Was your child angry? |  |  |  |
| Was it hard to take your child out in public? |  |  |  |
| Was it hard to comfort your child? |  |  |  |
| Was it hard to know what your child needs? |  |  |  |
| Was it hard to keep your child on a schedule or routine? |  |  |  |
| Was it hard to get your child to obey you? |  |  |  |

**2.** DURING the lockdown, your child:

|  | Not at all | Somewhat | Very Much |
| --- | --- | --- | --- |
| Does your child seem nervous or afraid? |  |  |  |
| Does your child seem sad or unhappy? |  |  |  |
| Does your child get upset if things are not done in a certain way? |  |  |  |
| Does your child have a hard time with change? |  |  |  |
| Does your child have trouble playing with other children? |  |  |  |
| Does your child break things on purpose? |  |  |  |
| Does your child fight with other children? |  |  |  |
| Does your child have trouble paying attention? |  |  |  |
| Does your child have a hard time calming down? |  |  |  |
| Does your child have trouble staying with one activity? |  |  |  |
| Is your child aggressive? |  |  |  |
| Is your child fidgety or unable to sit still? |  |  |  |
| Is your child angry? |  |  |  |
| Is it hard to take your child out in public? |  |  |  |
| Is it hard to comfort your child? |  |  |  |
| Is it hard to know what your child needs? |  |  |  |
| Is it hard to keep your child on a schedule or routine? |  |  |  |
| Is it hard to get your child to obey you? |  |  |  |

**Range 6-18 years**

**1.** In the following questions indicate if you have observed a different frequency of the following behaviors of your child during the lockdown due to the COVID-19 pandemic than in the past.

|  | Never | Sometimes | Often |
| --- | --- | --- | --- |
| Complains of aches and pains |  |  |  |
| Spends more time alone |  |  |  |
| Tires easily, has little energy |  |  |  |
| Fidgety, unable to sit still |  |  |  |
| Has trouble with teacher |  |  |  |
| Less interested in school |  |  |  |
| Acts as if driven by a motor |  |  |  |
| Daydreams too much |  |  |  |
| Distracted easily |  |  |  |
| Is afraid of new situations |  |  |  |
| Feels sad, unhappy |  |  |  |
| Is irritable, angry |  |  |  |
| Feels hopeless |  |  |  |
| Has trouble concentrating |  |  |  |
| Less interested in friends |  |  |  |
| Fights with other children |  |  |  |
| Absent from school |  |  |  |
| School grades dropping |  |  |  |
| Is down on him or herself |  |  |  |
| Visits the doctor with doctor finding nothing wrong |  |  |  |
| Has trouble sleeping |  |  |  |
| Worries a lot |  |  |  |
| Wants to be with you more than before |  |  |  |
| Feels he or she is bad |  |  |  |
| Takes unnecessary risks |  |  |  |
| Gets hurt frequently |  |  |  |
| Seems to be having less fun |  |  |  |
| Acts younger than children his or her age |  |  |  |
| Does not listen to rules |  |  |  |
| Does not show feelings |  |  |  |
| Does not understand other people’s feelings |  |  |  |
| Teases others |  |  |  |
| Blames others for his or her troubles |  |  |  |
| Takes things that do not belong to him or her |  |  |  |
| Refuses to share |  |  |  |

Questions reserved for the child

*In the following questions the direct participation of the child / adolescent is required. If your child is having trouble responding, please answer yourself, choosing the option that you think best suits your child.*

**1.** How worried are you about being infected with the COVID-19?

- I'm very worried

- I'm quite worried

- I'm slightly worried

- I'm not worried

**2.** How optimistic are you about the end of the epidemic?

- I'm very optimistic

- I'm quite optimistic

- I'm slightly optimistic

- I'm not optimistic

**3.** For each of the following sentences indicate the one that best represents you, referring to the LAST PERIOD since you were at home.

|  | Not At All | A Little | Some | A Lot |
| --- | --- | --- | --- | --- |
| I was bothered by things that usually don’t bother me |  |  |  |  |
| I did not feel like eating, I wasn’t very hungry |  |  |  |  |
| I wasn’t able to feel happy, even when my family or friends tried to help me feel better |  |  |  |  |
| I felt like I was just as good as other kids |  |  |  |  |
| I felt like I couldn’t pay attention to what I was doing |  |  |  |  |
| I felt down and unhappy |  |  |  |  |
| I felt like I was too tired to do things |  |  |  |  |
| I felt like something good was going to happen |  |  |  |  |
| I felt like things I did before didn’t work out right |  |  |  |  |
| I felt scared. |  |  |  |  |
| I didn’t sleep as well as I usually sleep |  |  |  |  |
| I was happy |  |  |  |  |
| I was more quiet than usual |  |  |  |  |
| I felt lonely, like I didn’t have any friends |  |  |  |  |
| I felt like kids I know were not friendly or that hey didn’t want to be with me. |  |  |  |  |
| I had a good time |  |  |  |  |
| I felt like crying |  |  |  |  |
| I felt sad |  |  |  |  |
| I felt people didn’t like me |  |  |  |  |
| It was hard to get started doing things |  |  |  |  |

**4.** For each of the following sentences indicate the one that best represents you, referring to the LAST PERIOD since you were at home.

|  | Not True  or Hardly  Ever True | Somewhat  True or  Sometimes  True | Very True  or Often  True |
| --- | --- | --- | --- |
| When I feel frightened, it is hard to breathe |  |  |  |
| I get headaches when I am at school |  |  |  |
| I don’t like to be with people I don’t know well |  |  |  |
| I get scared if I sleep away from home |  |  |  |
| I worry about other people liking me |  |  |  |
| When I get frightened, I feel like passing out |  |  |  |
| I am nervous |  |  |  |
| I follow my mother or father wherever they go |  |  |  |
| People tell me that I look nervous |  |  |  |
| I feel nervous with people I don’t know well |  |  |  |
| I get stomachaches at school |  |  |  |
| When I get frightened, I feel like I am going crazy |  |  |  |
| I worry about sleeping alone |  |  |  |
| I worry about being as good as other kids |  |  |  |
| When I get frightened, I feel like things are not real |  |  |  |
| I have nightmares about something bad happening to my parents |  |  |  |
| I worry about going to school |  |  |  |
| When I get frightened, my heart beats fast |  |  |  |
| I get shaky |  |  |  |
| I have nightmares about something bad happening to me |  |  |  |
| I worry about things working out for me |  |  |  |
| When I get frightened, I sweat a lot |  |  |  |
| I am a worrier |  |  |  |
| I get really frightened for no reason at all |  |  |  |
| I am afraid to be alone in the house |  |  |  |
| It is hard for me to talk with people I don’t know well |  |  |  |
| When I get frightened, I feel like I am choking |  |  |  |
| People tell me that I worry too much |  |  |  |
| I don’t like to be away from my family |  |  |  |
| I am afraid of having anxiety (or panic) attacks |  |  |  |
| I worry that something bad might happen to my parents |  |  |  |
| I feel shy with people I don’t know well |  |  |  |
| I worry about what is going to happen in the future |  |  |  |
| When I get frightened, I feel like throwing up |  |  |  |
| I worry about how well I do things |  |  |  |
| I am scared to go to school |  |  |  |
| I worry about things that have already happened |  |  |  |
| When I get frightened, I feel dizzy |  |  |  |
| I feel nervous when I am with other children or adults and I have to do  something while they watch me (for example: read aloud, speak, play a  game, play a sport) |  |  |  |
| I feel nervous when I am going to parties, dances, or any place where there  will be people that I don’t know well |  |  |  |
| I am shy |  |  |  |

Do you have other children?

- Yes  Child 2

- No  Send questionnaire

* this specific question is present only in the survey dedicated to children with neuropsychiatric disorders.
